# A Non-Coding RNA Within the *Rasgrf1* Locus in Mouse Is Imprinted and Regulated by Its Homologous Chromosome in *Trans*

**DOI:** 10.1371/journal.pone.0013784

**Published:** 2010-11-02

**Authors:** Chelsea M. Brideau, Krista P. Kauppinen, Rebecca Holmes, Paul D. Soloway

**Affiliations:** Division of Nutritional Sciences, College of Agriculture and Life Sciences, Cornell University, Ithaca, New York, United States of America; Victor Chang Cardiac Research Institute, Australia

## Abstract

**Background:**

*Rasgrf1* is imprinted in mouse, displaying paternal allele specific expression in neonatal brain. Paternal expression is accompanied by paternal-specific DNA methylation at a differentially methylated domain (DMD) within the locus. The *cis*-acting elements necessary for *Rasgrf1* imprinting are known. A series of tandem DNA repeats control methylation of the adjacent DMD, which is a methylation sensitive enhancer-blocking element. These two sequences constitute a binary switch that controls imprinting and represents the Imprinting Control Region (ICR). One paternally transmitted mutation, which helped define the ICR, induced paramutation, in *trans*, on the maternal allele. Like many imprinted genes, *Rasgrf1* lies within an imprinted cluster. One of four noncoding transcripts in the cluster, *AK015891*, is known to be imprinted.

**Methodology/Principal Findings:**

Here we demonstrate that an additional noncoding RNA, *AK029869*, is imprinted and paternally expressed in brain throughout development. Intriguingly, any of several maternally inherited ICR mutations affected expression of the paternal *AK029869* transcript in *trans*. Furthermore, we found that the ICR mutations exert different *trans* effects on *AK029869* at different developmental times.

**Conclusions/Significance:**

Few *trans* effects have been defined in mammals and, those that exist, do not show the great variation seen at the *Rasgrf1* imprinted domain, either in terms of the large number of mutations that produce the effects or the range of phenotypes that emerge when they are seen. These results suggest that *trans* regulation of gene expression may be more common than originally appreciated and that where *trans* regulation occurs it can change dynamically during development.

## Introduction

Genes inherited in two copies, one from each parent, are normally functionally equivalent, however, notable exceptions to this have been known for many years [1]–[5]. Among the genes that are functionally distinguishable according to their parent of origin are the imprinted genes, for which one parental copy is silenced while the other is expressed. Because these genes have unique modes of regulation and are often implicated in disease syndromes, much effort has been devoted to their identification and study.

The first imprinted locus identified was an artificial transgene carrying elements of the RSV LTR and a translocated c-myc gene. This transgene was expressed in the heart, but only when inherited from the father. Furthermore, the silencing that occurred upon maternal transmission was accompanied by DNA methylation of the transgene, which has been known to regulate gene expression [6]. Shortly after this discovery, two naturally occurring genes, *H19* and *Igf2r*, were shown to exhibit parent of origin specific expression [7], [8] and DNA methylation [9], [10]. Since these discoveries, the role of DNA methylation in the regulation of imprinted genes was firmly established [11]. Besides DNA methylation, additional factors are known to regulate imprinted gene expression, including the covalent modifications to histone proteins associated with DNA [12]–[16], the CTCF protein, [17]–[22], and physical interactions between the two parental chromosomes [23]–[28].

**Figure 1:**
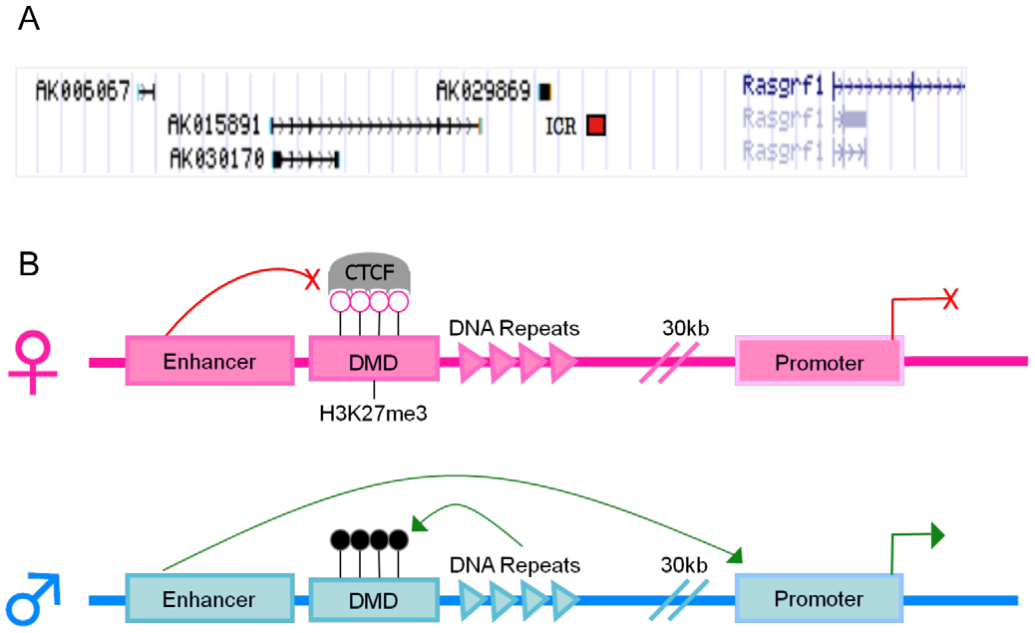
Organization and regulation of the *Rasgrf1* imprinted region. (A) UCSC Genome Browser depiction of the *Rasgrf1* imprinted region on mouse chromosome 9. Transcripts from *Rasgrf1* itself and four non-coding genes (*AK006067, AK030170, AK029869, AK015891*) are shown. The ICR consisting of the DMD and adjacent tandem repeats lie 30 kbp upstream of *Rasgrf1*. (B) Model for *Rasgrf1* imprinting. Tandem repeats program establishment and maintenance of DNA methylation on the paternal (blue) DMD [32], [33], which blocks H3K27me3 placement [14] as well as CTCF binding and enhancer blocking activities of the DMD, allowing enhancer-driven expression [22]. Tandem repeats stimulate H3K27me3 on the maternal DMD (pink), which blocks DNA methylation [14], allowing CTCF binding and enhancer-blocker activities, which silences *Rasgrf1* transcription [22]. Black and white circles over the DMD respectively depict methylated and unmethylated CpGs.

DNA sequences that control imprinting lie within the ICR, which often regulates imprinting of several nearby genes. *Rasgrf1* is paternally expressed in brain [29] and its ICR is located 30 kb upstream of the *Rasgrf1* transcription start site between *Rasgrf1* and four upstream non-coding (nc) RNAs (Figure 1A). One of these ncRNAs, *A19* (also called *AK015891*), located ∼10 kb upstream of the *Rasgrf1* ICR, is also imprinted in brain, where expression is solely from the paternal allele [30]. As ICRs have only been identified for seven imprinted clusters [31], the *Rasgrf1* system provides an uncommon opportunity to study regulation of imprinted gene expression.

The *Rasgrf1* ICR contains a differentially methylated domain (DMD) as well as a series of tandem DNA repeats, consisting of 40 copies of a 41 bp repeat unit. The DMD acquires DNA methylation exclusively on the paternal allele and placement of DNA methylation is controlled by the tandem repeats (Figure 1B) [32], [33]. The repeats are required for both establishment and maintenance of allele-specific DNA methylation during spermatogenesis and through pre-implantation development. Only a few other sequences have been implicated as *cis*-acting regulators of DNA methylation [34]–[36]. Imprinted expression is a result of both allele-specific binding of the methylation-sensitive enhancer blocker-binding protein, CTCF, and allele-specific DNA methylation. CTCF binds the unmethylated maternal DMD and disrupts enhancer to promoter interaction, resulting in silencing of *Rasgrf1*. On the paternal allele, the DMD is methylated, preventing binding of CTCF and allowing paternal allele expression [22]. The presence of the DNA repeats on the paternal allele is necessary for the establishment and for the maintenance of DNA methylation at the DMD until implantation at embryonic day 5.5 (e5.5). Deletion of the paternal repeats before this time point leads to loss of DNA methylation on the paternal allele, resulting in silencing of *Rasgrf1* [33]. The DNA repeats, in combination with the DMD, constitute a binary switch that is necessary for imprinted expression of *Rasgrf1*.

We previously generated a *Rasgrf1* mutation in which the *Rasgrf1* repeats were replaced with sequences referred to as Region 2 (R2) that were implicated in the control of *Igf2r* imprinting [37]. When paternally inherited, this modification allowed both methylation and *Rasgrf1* expression, in *cis*, of the paternal allele, indicating R2 could substitute for the endogenous *Rasgrf1* repeats. Interestingly, the wild-type maternal allele also became methylated and expressed in *trans*, and once modified, the altered epigenetic state persisted in the next generation, an example of paramutation [37]. This was our first observation of a *trans*-expression effect in the *Rasgrf1* genomic region.

Here, we report that a second ncRNA, *AK029869*, located ∼5 kb upstream of the *Rasgrf1* ICR is imprinted and paternally expressed in neonatal brain. We also show that deletion of the tandem repeats within the *Rasgrf1* ICR perturbs imprinting of *AK029869*, and has the ability to do so in *trans*. Finally, we demonstrate that several ICR mutations on the maternal copy of *Rasgrf1* lead to silencing of the normally expressed *AK029869* paternal allele in *trans*. These constitute additional examples of *trans*-expression effects within the *Rasgrf1* imprinted cluster and demonstrate the robust communication between *Rasgrf1* alleles across chromosomal boundaries.

## Results

### *AK029869* is imprinted in brain

**Figure 2:**
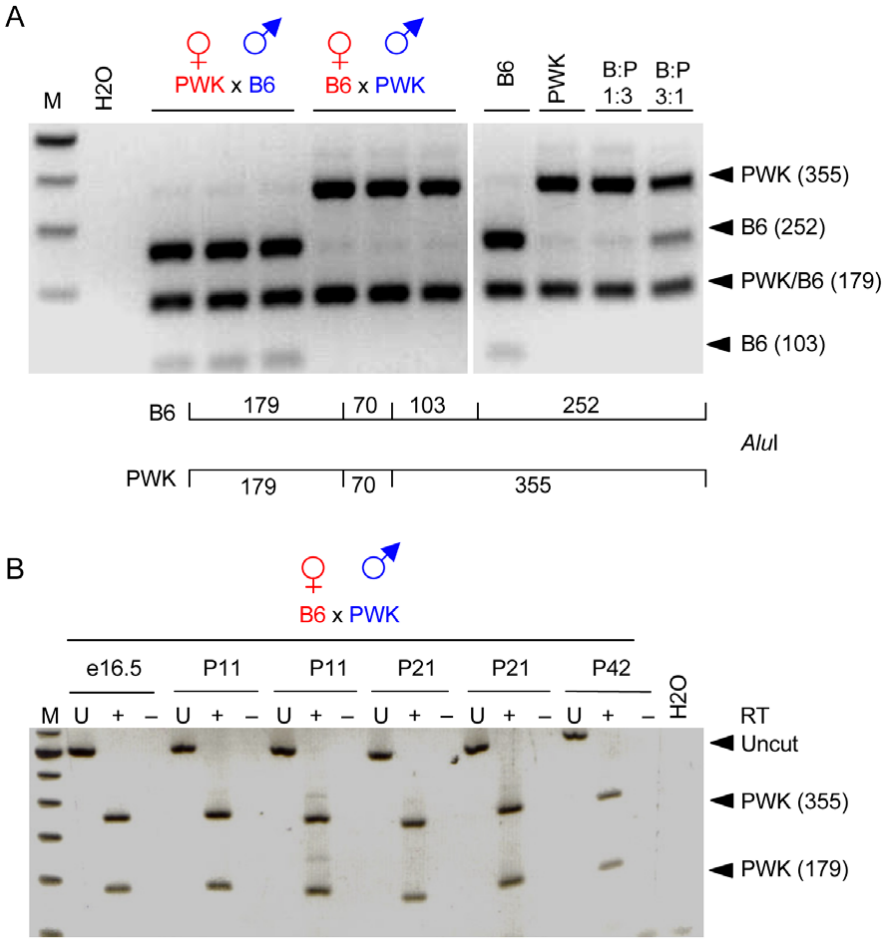
*AK029869* is imprinted in brain at many developmental stages. (A) Brains from post-natal day 10 (P10) or (B) embryonic day 16.5 (e16.5) mice and older (P11–P42) were assayed for *AK029869* imprinted expression. Animals were progeny of C57BL/6 (B6) and PWK parents that carry the *Alu*I polymorphism in *AK029869* shown below the gel in (A), where numbers represent *Alu*I fragment sizes in nucleotides. Maternal strain is shown first in red followed by paternal strain in blue. Where no cross is shown, the mice were inbred. (A) RT-PCR products from six independent progeny of two reciprocal crosses were digested with *Alu*I before gel analysis (left panel). Inbred strains and mixtures of inbred cDNAs in the ratios indicated were included as markers and controls for amplification bias (right panel). Band sizes and the strain source are to the left of the gel. (B) cDNAs were analyzed as in (A) with the exception that undigested (U) RT-PCR products were also analyzed and PCR was done using mRNAs that were (+) or were not (–) reverse transcribed (RT). M identifies marker lanes.

To test the imprinting status of *AK029869*, we first identified useful SNPs between the polymorphic mouse strains PWK and C57BL/6 (B6) to distinguish the expression from the two alleles. We chose one SNP that overlapped with an *Alu*I restriction site. *Alu*I digestion produces distinct banding patterns for the two strains. We established reciprocal crosses between PWK and B6, extracted RNA from brains of post partum day 10 (P10) progeny and then performed RT-PCR followed by *Alu*I digestion to identify the expressed alleles. We found that *AK029869* expression was exclusively from the paternal allele at P10 (Figure 2A). Though there was a bias in the efficiency of amplification of the two alleles, paternal allele specific expression was quite clear. As imprinted expression can be developmental time point specific, we repeated the analysis using brain samples collected between embryonic day 16.5 (e16.5) through P42. We found that *AK020869* was paternally expressed throughout this interval (Figure 2B). In addition to brain, we performed the analysis on testes taken between embryogenesis and adulthood. *AK029869* expression was biallelic in testes at all stages tested (not shown).

### Allele-specific expression of *AK029869* depends on the *Rasgrf1* repeats

**Figure 3:**
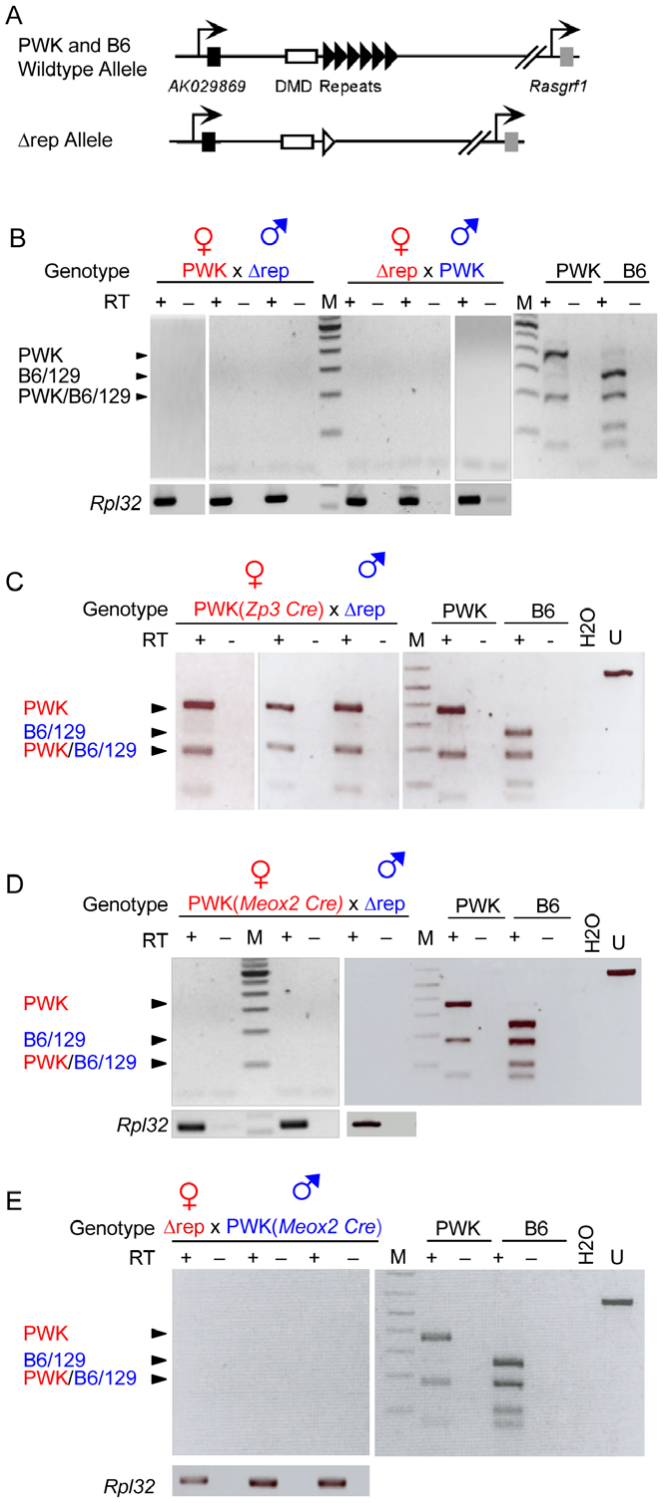
Normal imprinted *AK029869* expression requires the *Rasgrf1* tandem DNA repeats on both alleles at different stages of development. (A) The wild-type *Rasgrf1* allele (top) includes a 1.6 kbp repeat element (black triangles) needed to program DNA methylation at the DMD (white rectangle). In the repeat deficient allele (bottom), the repeats were replaced with a single loxP site (white triangle). The repeat deficient allele was prepared as a germ line mutation [32] and as a conditional allele whereby the repeats can be deleted by Cre recombinase [33]. Deletion of the repeats from the conditional allele leaves an frt site adjacent and in addition to the loxP site. (B) Allele-specific expression of *AK029869* was assayed as in Figure 2 using P10 brain cDNAs from progeny of reciprocal crosses between wild-type PWK animals and B6 mice carrying the repeat deletion in their germ line. The mutation was made on a 129S4Jae background, which has the same polymorphism as B6. The analysis was repeated using mice from which the repeats were removed by *Cre* transgenes at different times after fertilization. (C) Paternal repeats were deleted by a maternally transmitted *Zp3-Cre* transgene that can delete the repeats upon fertilization [38] or (D) by a maternally transmitted *Meox2-Cre* transgene that can delete the paternal repeats in the embryonic ectoderm of the e5.5 epiblast [39]. (E) The maternal repeats were deleted by a paternally transmitted *Meox2-Cre* transgene. Animals that lacked complete deletion of the repeats were excluded from the analysis (not shown). *Rpl32* was included as a control for samples lacking detectable *AK029869* expression. The strain origins of bands are shown to the left of the gels with the maternal and paternal strains shown in red and blue, respectively.

Next, we wanted to test whether paternal allele-specific expression of *AK029869* depended upon the presence of the tandem DNA repeats in the ICR, as is the case for *Rasgrf1*. To do this, we established reciprocal crosses between PWK animals and mice carrying a targeted deletion of the tandem DNA repeats (Δrep, Figure 3A, [32]). The Δrep allele, and all other *Rasgrf1* ICR mutations described below were prepared on the 129S4Jae background, which shares the same *AK029869 Alu*I site polymorphism as B6. As with *Rasgrf1*, paternal inheritance of a Δrep allele led to silencing, in *cis*, of *AK029869*. Expression of the normally silent maternal allele was unaffected, as was DNA methylation at the maternal DMD [32]. Unexpectedly, maternal inheritance of the Δrep allele led to silencing, in *trans*, of the paternal *AK029869* allele (Figure 3B). This *trans*-silencing was independent of DNA methylation, as maternal inheritance of a Δrep allele did not change the DNA methylation status of either DMD, or expression of *Rasgrf1* [32].

### Effect of timing of repeat deletion on *AK029869* expression

**Figure 4:**
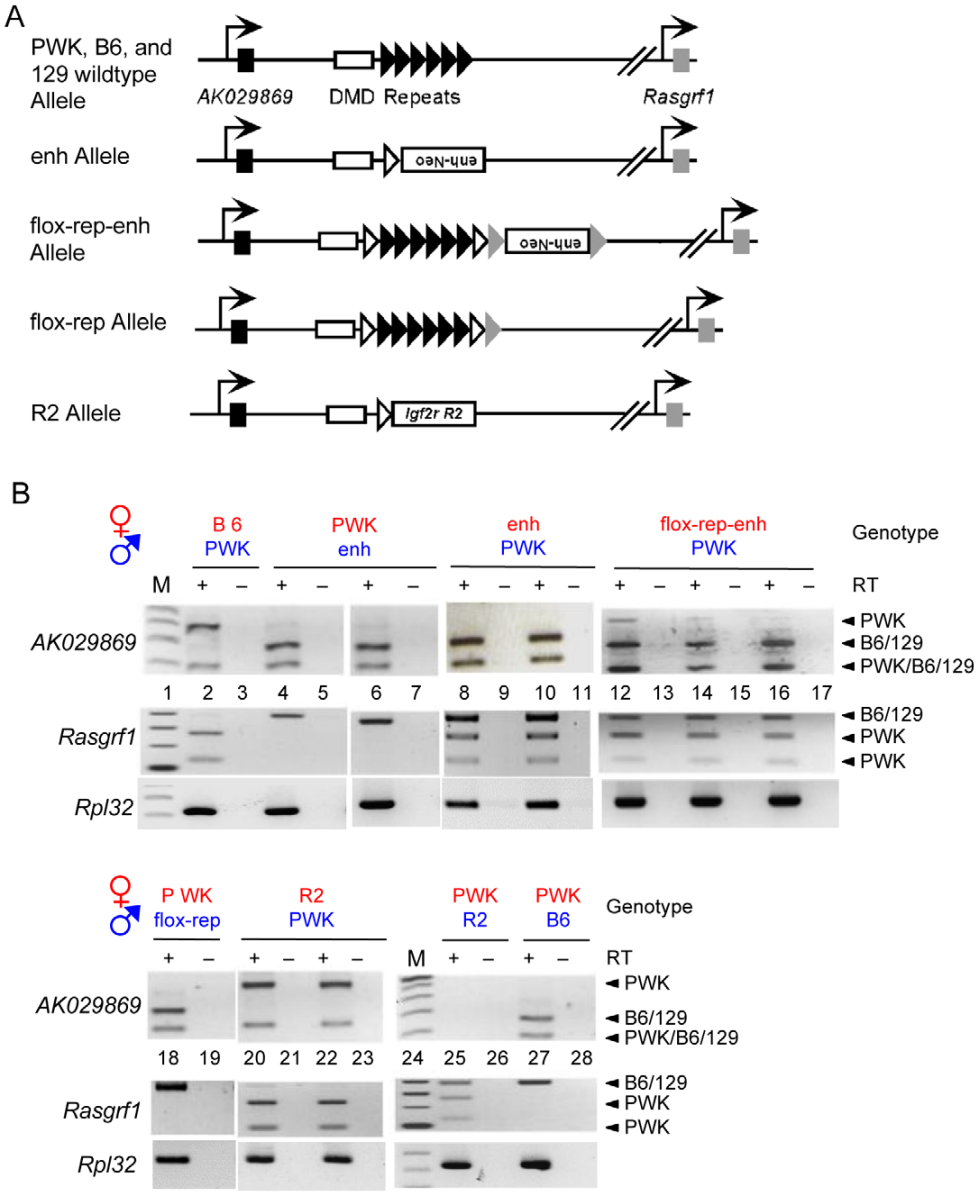
*Trans* control of *AK029869* by several *Rasgrf1* ICR mutations. (A) Structure of the wild-type *Rasgrf1* allele and of several additional ICR mutations including enh [22], flox-rep-enh [33], flox-rep [33] and R2 [37]. Enh-neo is a neomycin phosphotransferase cassette carrying the PGK enhancer; *Igf2 R2* is the Region 2 DMR from *Igf2r*; black, white and grey triangles respectively depict wild-type repeats, loxP sites, and frt sites. All mutated alleles were on the 129 background, which shares the same *Alu*I restriction polymorphism with B6. (B) Allele-specific expression of *AK029869*, and total expression of *Rpl32* were assayed as in Figure 2 using brain cDNAs from P10 progeny of reciprocal crosses between PWK mice and mates carrying various germ line mutations. Allele specific analysis of *Rasgrf1* expression was as described [29].

After fertilization, there is a developmental time before which the repeats are required to maintain methylation at the *Rasgrf1* DMD, and after which they are dispensable [33]. Therefore, we wondered if the *cis*- or *trans*-silencing we observed at P10 in *Rasgrf1* repeat-deficient mice was also sensitive to the developmental stage at which the repeats were deleted. To control deletion of the maternal or paternal repeats after fertilization, we used mice homozygous for an allele with loxP sites flanking the *Rasgrf1* DNA repeats (flox-rep, Figure 4, [33]). This allele is functionally wild-type, preserving normal imprinted expression of both *AK029869* and *Rasgrf1* as well as imprinted DNA methylation [33]. We crossed mice bearing the flox-rep allele with different *Cre* transgenes to delete the repeats. To facilitate allele-specific expression analysis, we bred the *Cre* transgenes onto the PWK mouse background. The breeding strategy is shown in Figure S1. We used the *Zp3 Cre* transgene (Figure 3C), which is active at e0.0 and can delete the repeats at the one-cell stage [38], and the *Meox2 Cre* transene (Figure 3D, E), which is active at e5.5 and deletes the repeats in the embryonic ectoderm [39]. Depending on the direction of the cross, we were able to delete the repeats at these two time points from the maternally or the paternally transmitted allele.

From these crosses, we collected P10 brain and testes from 50 litters of mice. The potentially informative animals were those bearing the *Cre* transgene, one 129S4Jae allele and one PWK allele at *AK029869*. We also genotyped each tissue sample to determine the extent of deletion of the loxP-flanked *Rasgrf1* repeats because only mice that had complete *Cre*-mediated deletion of the repeats were informative. We prepared cDNA from all informative brain samples and performed allele-specific expression analysis of *AK029869*.

This analysis revealed multiple patterns of *AK029869* expression control by the *Rasgrf1* repeats that changed according to developmental stage. When the paternal repeats were deleted by the *Zp3 Cre* transgene near the time of fertilization, *AK029869* expression was both silenced in *cis* on the paternal allele and activated in *trans* on the maternal allele (Figure 3C). Interestingly, this pattern changed when the paternal repeats were deleted later in the embryonic ectoderm of the e5.5 epiblast by a maternal *Meox2 Cre* transgene. Deletion at this later stage caused only paternal allele silencing but no maternal allele activation (Figure 3D). When the maternal repeats were deleted by a paternally transmitted *Meox2 Cre* transgene, paternal expression of *AK029869* expression was silenced in *trans* (Figure 3E). As *Zp3 Cre* is not expressed in sperm, it could not be used to delete the maternal repeats upon fertilization. These results demonstrated that germ line inheritance of a *Rasgrf1* repeat deletion allele silenced the normally active paternal allele in *cis* and in *trans*, whereas somatic deletion of the repeats after fertilization produced multiple *cis* and *trans* regulatory effects that included silencing and activation and that varied by developmental stage.

### Additional *Rasgrf1* ICR mutations result in *cis* and *trans* silencing of *AK029869*

**Figure 5:**
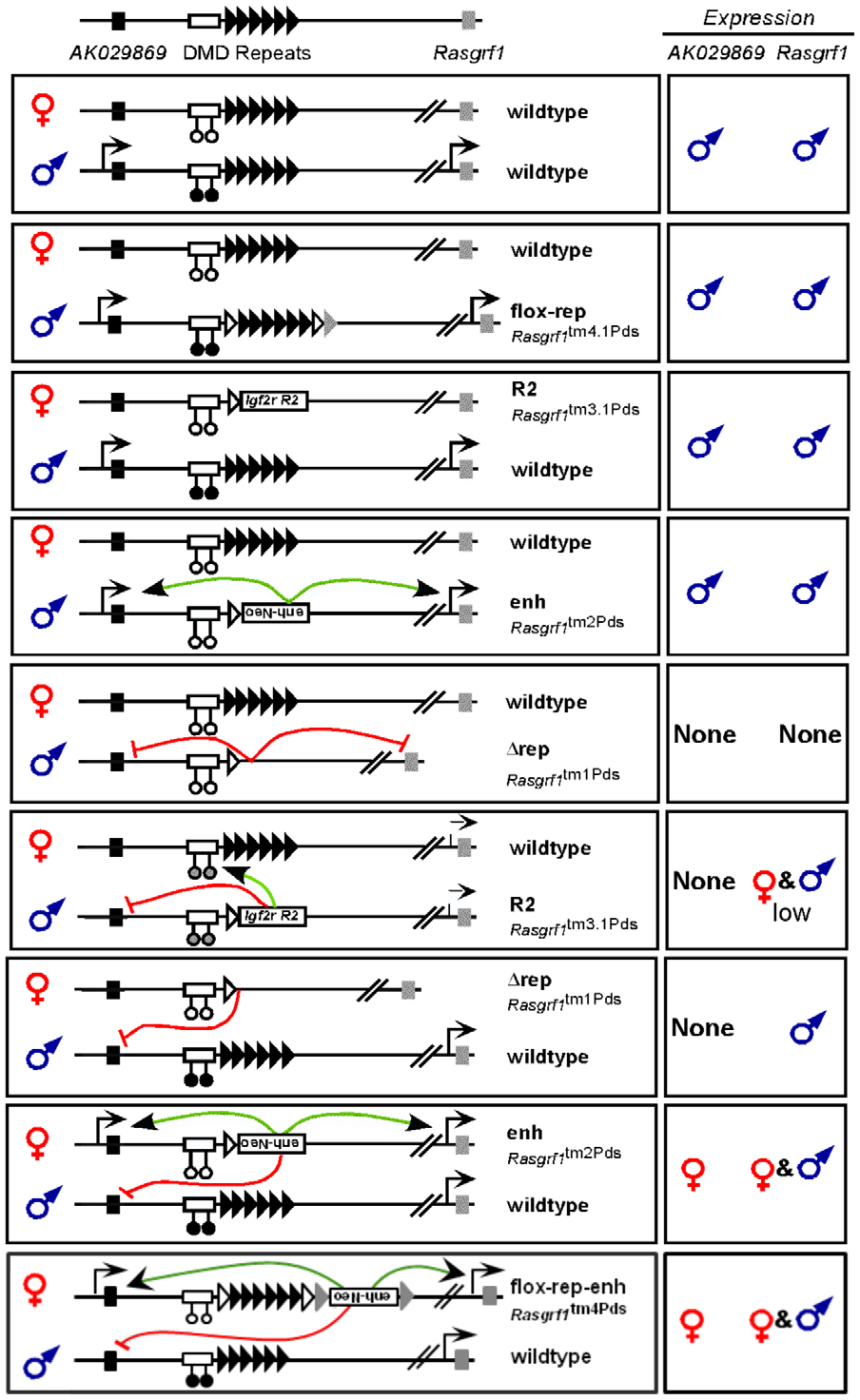
Summary of *Rasgrf1* and *AK029869* expression phenotypes of ICR mutants. Each of the allele combinations represented in rectangles on the left hand side were tested for their effect on both *AK029869* and *Rasgrf1* expression. Abbreviated names for the alleles used here as well as their formal names are shown. Green and red lines respectively indicate positive or negative effects on expression that do not occur in wild-type mice. The ♂ and ♀ symbols in rectangles on the right hand side indicate whether expression, if any, is from the maternal (♀) or the paternal (♂) alleles. Black circles below the DMD indicate that the DMD is methylated and white circles indicate lack of methylation. Grey circles and dashed lines in mice with a paternal R2 allele indicate partial methylation and expression.

In addition to the Δrep allele, we tested several other ICR alleles for their ability to influence *AK029869* imprinting, when transmitted as germ line mutations. Figure 4 shows the alleles, their effects on *AK029869* expression, and Figure 5 summarizes the results.

The first additional ICR mutation had an extra enhancer (enh) in place of the tandem DNA repeats [22]. When paternally transmitted, expression of *AK029869* was from the paternal allele, as it is in wild-type mice (Figure 4B, lanes 4–7). However, when maternally transmitted, the paternal allele was silenced in *trans* and the maternal allele was inappropriately activated in *cis* (Figure 4B, lanes 8–11). The second additional ICR mutation (flox-rep-enh) carried loxP sites on either side of the repeats, with an extra enhancer 3′ of the repeats [33]. Like the enh allele, maternal transmission inappropriately activated the maternal allele in *cis*, and expression of the paternal allele was strongly diminished in *trans* (Figure 4, lanes 12–17).

The extra enhancer on the enh and flox-rep-enh alleles was from the housekeeping gene *Pgk* and is known to cause over expression of *Rasgrf1* [40]. We wondered whether the enhancer was so strong that it boosted *AK029869* expression from the mutated allele high enough to mask otherwise normal levels of expression from the homologous wild-type PWK allele. This could lead us to conclude, erroneously, that maternal transmission of the mutated enhancer carrying alleles silenced the paternal allele in *trans*. To explore this possibility, we mixed brain cDNAs from a wild-type PWK mouse and a mouse with the enh allele in varying ratios and looked for evidence of amplification of both alleles. In each case, even when the mixture contained a three fold excess of cDNA from enh-bearing mice, we were able to easily detect PCR products diagnostic of both alleles. This provides confidence that PCR artifacts did not confound our conclusion that maternally transmitted extra enhancer alleles silenced the paternal allele in *trans* (Figure S2).

The third additional ICR mutation we tested lacked the *Rasgrf1* repeats and replaced them with the differentially methylated domain, referred to as Region 2, from the imprinted and maternally expressed *Igf2r* gene (R2 allele). When the R2 allele was maternally inherited, *AK029869* demonstrated normal expression from the paternal allele only (Figure 4, lanes 20–23). However, when paternally inherited, the R2 allele silenced the paternal allele in *cis*. Interestingly, this differed from the effect of the paternal R2 allele on *Rasgrf1* expression. When the R2 allele was paternally inherited, the paternal copy of *Rasgrf1* was activated in *cis* and the normally silent maternal *Rasgrf1* allele was activated in *trans* ([37] and Figure 4, lanes 25–26). This is because Region 2 positively regulates DNA methylation at the *Rasgrf1* DMD, in both *cis* and *trans,* when paternally inherited. A caveat is that paternal *Rasgrf1* expression is reduced, but still detectable, with an R2 paternal allele [37]. Because *AK029869* expression is low in wild-type animals, it is possible that paternal transmission of the R2 allele reduced *AK029869* expression below the threshold of detection. Maternal transmission of the R2 allele has no effect on *Rasgrf1* expression or DNA methylation at the DMD [37].

## Discussion

Although the role of DNA methylation in the control of imprinted expression is relatively well understood, advances in the understanding of other factors regulating imprinted expression have been made more slowly. The body of evidence for the involvement of *trans*-expression effects in imprinted gene regions is growing. At the imprinted gene, *Ins2*, a mutation on the paternal allele silenced expression of the normally expressed maternal allele in *trans* [41]. A mutation to the paternal allele of the paternally expressed gene, *Snrpn*, activated expression of the normally silenced maternal allele in *trans* [42]. At a nearby locus, a mutation on the maternal allele of the maternally expressed gene *Ube3a*, silenced expression of the paternally expressed antisense transcript, *Ube3a-ATS*, in *trans* [43]. At the *H19/Igf2* imprinted locus, a maternal allele deletion at the *H19* allele led to a reduction in the level of DNA methylation at the wild-type *Igf2* paternal allele [44]. Also at the *H19/Igf2* locus, replacement of a differentially methylated domain between *Ins2* and *Igf2* on the paternal allele led to activation of the normally silent *Igf2* maternal allele [45]. Similarly, when this mutation was present on the maternal allele, the normally silent *H19* paternal allele was activated [45]. Consistent with this *trans* chromosomal communication that affects expression at imprinted genes is evidence that physical interactions occur between imprinted loci and both homologous and non-homologous loci [23], [27].

In addition to imprinted genes, *trans*-effects occur at other loci including the X-chromosome inactivation center [46], odorant receptors [47], and T-helper cytokines [48]. As with imprinted genes, the expression of these genes is also epigenetically regulated [49]–[51]. During mammalian X chromosome inactivation, one of the female X chromosomes is silenced to effect equal levels of X chromosome gene expression between XX and XY individuals. X chromosome inactivation is regulated by the genes *Xite*, *Tsix*, and *Xist.* Two of these genes, *Tsix* and *Xite*, also function in *trans* to regulate X chromosome counting and choice through physical pairing of the two X chromosome homologues [46], [52]. Similar interactions occur between non-homologous chromosomes in T-helper cytokine gene expression and allelic exclusion of odorant receptor expression [47], [48], however, the significance of these interactions for gene expression are not clear [53]. Recent data using the Hi-C method show such *trans* chromosomal interactions are quite common in humans [54]. Little is known about the significance of these long-range genomic interactions or if they are responsible, mechanistically, for the *trans* expression effects we describe. The Hi-C data were collected for human cells and *Rasgrf1* is not imprinted in primates (K. Kauppinen, J.T. Brenna and PDS, unpublished) so it is not known what interactions occur with *Rasgrf1* in mouse, where it is imprinted.

The *Rasgrf1* imprinted cluster in mouse is uniquely positioned as a model to study *trans*-expression effects for several reasons. First, the *Rasgrf1* ICR has been well characterized. Sequences controlling DNA methylation have been identified [32] and the mechanism by which methylation controls expression has been determined [22]. Such details are lacking for other imprinted genes. Second, a variety of ICR mutation alleles are available for this region. Third, the effect that each of these alleles has on the methylation state of the ICR (and *Rasgrf1* imprinted expression) is known.

Here, we characterized a novel noncoding RNA within the *Rasgrf1* imprinting cluster, *AK029869*, and showed it is paternally expressed in brain and that this imprinting is subject to *trans* regulation by various ICR mutations. Imprinted expression of *AK029869* and the *trans* effects that regulate it are distinct in several ways from control of *Rasgrf1* imprinting, which we previously described [22], [32], [33], [37]. First, the tandem DNA repeats within the *Rasgrf1* ICR were important for proper imprinted expression of *AK029869*, as is the case for *Rasgrf1* expression. But whereas *Rasgrf1* imprinted expression depends only on the paternal repeats [32], proper *AK029869* imprinting depended on both parental repeats. Loss of either set silenced paternal expression of *AK029869*, with maternally deleted repeats causing silencing in *trans* (Figure 3B). Second, loss of the paternal *Rasgrf1* repeats upon fertilization caused silencing of *Rasgrf1* imprinted expression [33]. Strikingly, deletion of the paternal repeats during this time led to a reversal of imprinted expression of *AK029869* in brain, silencing the normally active paternal allele and activating the normally silent maternal allele (Figure 3C). Third, proper imprinted expression of *Rasgrf1* required the paternal repeats only prior to the epiblast stage. Deleting the repeats at the epiblast stage or later had no effect on *Rasgrf1* expression [33]. In contrast, when the repeats were deleted from either the maternal or paternal allele at the epiblast stage, the paternal allele was silenced (Figures 3D and 3E).

Regarding how the expression of *AK029869* is regulated, we considered three possibilities. We first considered that DNA methylation levels at the DMD might regulate *AK029869.* However, maternal inheritance of a Δrep allele, an enh allele, or a flox-rep-enh allele all preserved DNA methylation at the paternal DMD, but led to silencing in *trans* of the paternal *AK029869* allele ([22], [32], [33], Figures 3 and 4). This indicates that paternal DMD methylation is not sufficient for imprinted *AK029869* expression. In contrast, whenever the DMD was methylated, we observed expression of *Rasgrf1* [22], .

Second, we considered that proper imprinted expression of *AK029869* may occur as long as both the maternal and the paternal alleles carry some form of the tandem DNA repeats, but this is not the case. Mice inheriting a maternal copy of a flox-rep-enh allele had both the maternal and the paternal tandem DNA repeats, but underwent an inversion of the normal imprinting pattern, with the maternal allele becoming activated and the paternal allele becoming silenced. In addition, mice with a maternally inherited R2 allele lacked one copy of the DNA repeats but exhibited apparently proper paternal allele specific expression of *AK029869* (Figure 4).

Third, we considered that sequence spacing within the ICR might be critical for proper expression of *AK029869*. However, paternal inheritance of the R2 allele retained wild-type sequence spacing, as Region 2 and the tandem DNA repeats are both approximately 2kb, but this led to silencing in *cis* of the paternal allele (Figure 4). Therefore, in contrast to the binary switch model for expression of *Rasgrf1*, there is a distinct mechanism for *AK029869* imprinting that is far more complex than the relatively simple binary switch mechanism for *Rasgrf1* imprinting.

Despite the complexity of *AK029869* regulation, there are three patterns that emerged from our data. First, any changes in spacing within the ICR on the maternal allele led to silencing in *trans* of the paternal allele. For example, the R2 allele contains an approximately 2kb deletion of the tandem DNA repeats but, since Region 2 is roughly 2kb, insertion of Region 2 retains normal sequence spacing. When maternally inherited, the R2 allele allowed expression of the paternal allele. On the other hand, the flox-rep-enh allele changed the sequence spacing of the region, and led to paternal allele silencing. The one exception is the enh allele, which kept the maternal allele spacing but silenced the paternal allele in *trans*. The enhancer insertion may produce additional changes to local chromatin structure that over ride otherwise normal sequence spacing on the ICR.

A second pattern is that deletion of the paternal repeats, regardless of the resulting sequence spacing, led to silencing in *cis* of the paternal allele. For example, paternal inheritance of the R2 allele deleted the tandem DNA repeats and preserved sequence spacing, but silenced the paternal allele. Again, the one exception was if the repeats were replaced with the extra enhancer. The enh allele also deleted the tandem DNA repeats and preserved sequence spacing, but it allowed expression of the paternal allele. This represents the third pattern we observed, any allele with an extra enhancer led to activation of that allele in *cis*, regardless of sequence spacing or the presence of the repeats on that allele.

Therefore, it appears that sequence spacing in the region of the repeats, but not presence of the repeats, on the maternal allele is important, while the presence of the repeats, but not sequence spacing, on the paternal allele is important. Also, the presence of an extra enhancer may override these two requirements to allow expression in *cis*, but not in *trans*, indicating that access to an enhancer is necessary for expression of *AK029869*.

Other imprinted loci, including *Igf2*, undergo allele specific differences in three-dimensional conformation [24]–[26], [28]. One consequence of the maternal allele conformation is that it might prevent distant enhancers from interacting with the *Igf2* promoters, enforcing silence of the maternal copy of *Igf2*. This mechanism may control *Rasgrf1* imprinting. Additional and more complex CTCF dependent interchromosomal interactions occur between *Igf2* and other genes [27], and such regulation may be occurring to control *AK029869* imprinting. If this is the case, it could be dependent on sequence spacing, repeat-content, or a combination of the two, which could in turn be influenced by the presence of enhancers. Experiments such as 3C or FISH are needed to address this question. Nevertheless, the results discussed above clearly demonstrate an abundance of *trans*-expression effects within the *Rasgrf1* imprinted cluster.

## Materials and Methods

### SNP and restriction site identification

SNPs were identified in AK029869 using the Jackson Laboratory Mouse Genome Informatics website (http://www.informatics.jax.org). SNPs available between C57/BL6 and 129 versus PWK mouse strains were noted. In each case, potential SNPs were analyzed using NEB cutter (http://tools.neb.com/NEBcutter2/index.php) to select SNPs overlapping a restriction enzyme recognition site for identification of allele-specific expression.

### Ethics statement

The Institutional Animal Care and Use Committee at Cornell University approved all research involving animals described here, as required by the United States National Institutes of Health and Department of Agriculture. Cornell University is accredited by the Association for Assessment and Accreditation of Laboratory Animal Care.

### Tissue collection

Crosses were set up between either wild-type B6 and PWK mice or B6 mice homozygous for loxP-flanked copies of the *Rasgrf1* DNA repeats and PWK mice carrying either *Zp3 Cre* or *Meox2 Cre* alleles. In each case, crosses were set up as reciprocal pairs to either rule out expression differences due to strain QTLs or to examine the effect of inheritance of both maternal and paternal repeat deletions. The progeny of each of these crosses were sacrificed at P10 (except for the wild-type imprinting time course experiment) and a small portion of the brain was collected for genotyping. The remainder of the brain was snap frozen in liquid nitrogen for later use. P11 and younger mice were killed by decapitation; P21 and older animals were killed by CO_2_ asphyxiation.

### Genotyping

Brain DNA samples were prepared by lysing in Laird's lysis buffer plus proteinase K overnight followed by ethanol precipitation. Brain DNA was genotyped for the presence of one B6 allele and one PWK allele using primers either AKnewFWD (5′- CTT TCT CCA GCA ACC TAT C -3′) and AKnewREV (5′- AAG GAC CTG CCG CTT AAC T -3′) or primers PDS155 (5′- ATT CAC CGC TGC TGC TTA AA-3′ ) and AKR1-KPK (5′- TAG GAA AAT GGC TCG GTG TC -3′) for 40 cycles under the conditions 94°C for 30 seconds, 60°C for 1 minute, 72°C for 2 minutes. Also, for the repeat-deletion experiments, deletion of the DNA repeats was determined using the primer combination PDS16 (5′ - GCA CTT CGC TAC CGT TTC GC - 3′), PDS18 (5′ - TTT CTG CCA TCA TCC CAG CC - 3′), and PDS17 (5′ - TGT CCT CCA CCC CTC CAC C- 3′) and cycling conditions 94°C for 10 seconds, 61°C for 20 seconds, 72°C for 50 seconds for 40 cycles.

### RNA preparation

Brain samples were isolated from F1 progeny of reciprocal crosses at P10 (except in the case of the imprinting timecourse experiment) and total RNA was prepared. For each neonatal brain, 2 mls of GTC RNA lysis buffer was used (4M guanidium thiocyanate, 25 mM pH 7.0 sodium citrate, 100 mM beta-mercaptoethanol, 0.5% sarcosyl, 0.2M pH 4 sodium acetate, and 50% acidic phenol) and each brain was homogenized for 45 seconds at 18,000 rpm. Following homogenization, RNA was extracted with 0.2 volumes chloroform followed by isopropanol precipitation. RNA was resuspended in 10 mM Tris-EDTA.

### cDNA analysis

cDNA was prepared from 5 ug of RNA treated with 2.5 ul of DNaseI (Invitrogen). Amplification was done using random primers (Invitrogen) and Superscript II reverse transcriptase (Invitrogen). Following cDNA synthesis, nested PCR was performed using 0.5 ul cDNA as template. First round PCR was done with primers PDS155 (5′ - ATT CAC CGC TGC TGC TTA AA - 3′) and AKR1-KPK (5′ - TAG GAA AAT GGC TCG GTG TC - 3′) for 19 cycles. 2 ul of first round PCR product was diluted into 18 ul of water, and 1.5 ul of this dilution was used as template for 35 cycles of second round PCR. Second round PCR primers were AKnewFWD (5′ - CTT TCT CCA GCA ACC TAT C) and AKnewREV (5′ - AAG GAC CTG CCG CTT AAC T - 3′). In each case, cycling conditions were 94°C for 30 seconds, 60°C for 1 minute, 72°C for 2 minutes. 10 ul of second round PCR product was digested 5 hours to overnight with 1U *AluI* (NEB). Digests were heat inactivated and run on a 3% agarose gel.

## Supporting Information

Figure S1 Crossing scheme for developmental time point specific repeat deletions. To delete the DNA repeats as specific times after fertilization, we used an allele containing a loxP-flanked copy of the *Rasgrf1* DNA repeats in combination with the Cre transgenic mice. To facilitate allele-specific expression analysis, we bred specific Cre alleles onto the PWK/PhJ mouse background. These mice (PWK Cre) were mated with mice homozygous for a loxP-flanked version of the *Rasgrf1* DNA repeats (Floxed), which was created on the 129S4Jae strain background and backcrossed to C67BL/6. In the presence of Cre recombinase expression, the loxP-flanked repeats can be deleted at specific time points. Zp3 Cre is active at e0.0 and deletes the repeats at the one-cell stage, while Meox2 Cre is active at e5.5 and deletes the repeats in the embryonic ectoderm of the e5.5 epiblast around the time of implantation into the uterine wall. Depending on whether the loxP-flanked repeats were inherited maternally or paternally, we were able to delete the repeats at these time points on either the maternally or the paternally inherited allele. After genotyping to ensure that the animals were not mosaic for deletion of the DNA repeats, and to ensure that the animals carried one PWK and one 129S4Jae allele at the *AK029869* locus, we carried out allele-specific expression analysis.
(0.42 MB TIF)

Figure S2 The presence of an extra enhancer allele does not mask detection of expression from wild-type alleles in trans. cDNAs from a wildtype and an extra enhancer allele were mixed in varying ratios. The mixed cDNAs were subjected to allele specific expression analysis of *AK029869* as described in the main text. Strains and band sizes are shown to the right (see main text, Figure 2A). In each case, even when the extra enhancer allele (enh) was present in a 3∶1 ratio to the wild-type allele (WT), banding patterns from both alleles were present indicating that expression from the extra enhancer allele did not obscure expression from the wild-type allele during amplification. “ + ”  =  with reverse transcriptase, “ – ”  =  without reverse transcriptase, (-)  =  water, B6  =  C57Bl6/J, PWK  =  PWK/PhJ.
(0.17 MB TIF)
