# Supplementary figures and images for: A Non-Coding RNA Within the Rasgrf1 Locus in Mouse Is Imprinted and Regulated by Its Homologous Chromosome in Trans
Source: PLoS One. 2010 Nov 2;5(11):e13784. doi: 10.1371/journal.pone.0013784 (PMC2970558; doi:10.1371/journal.pone.0013784)

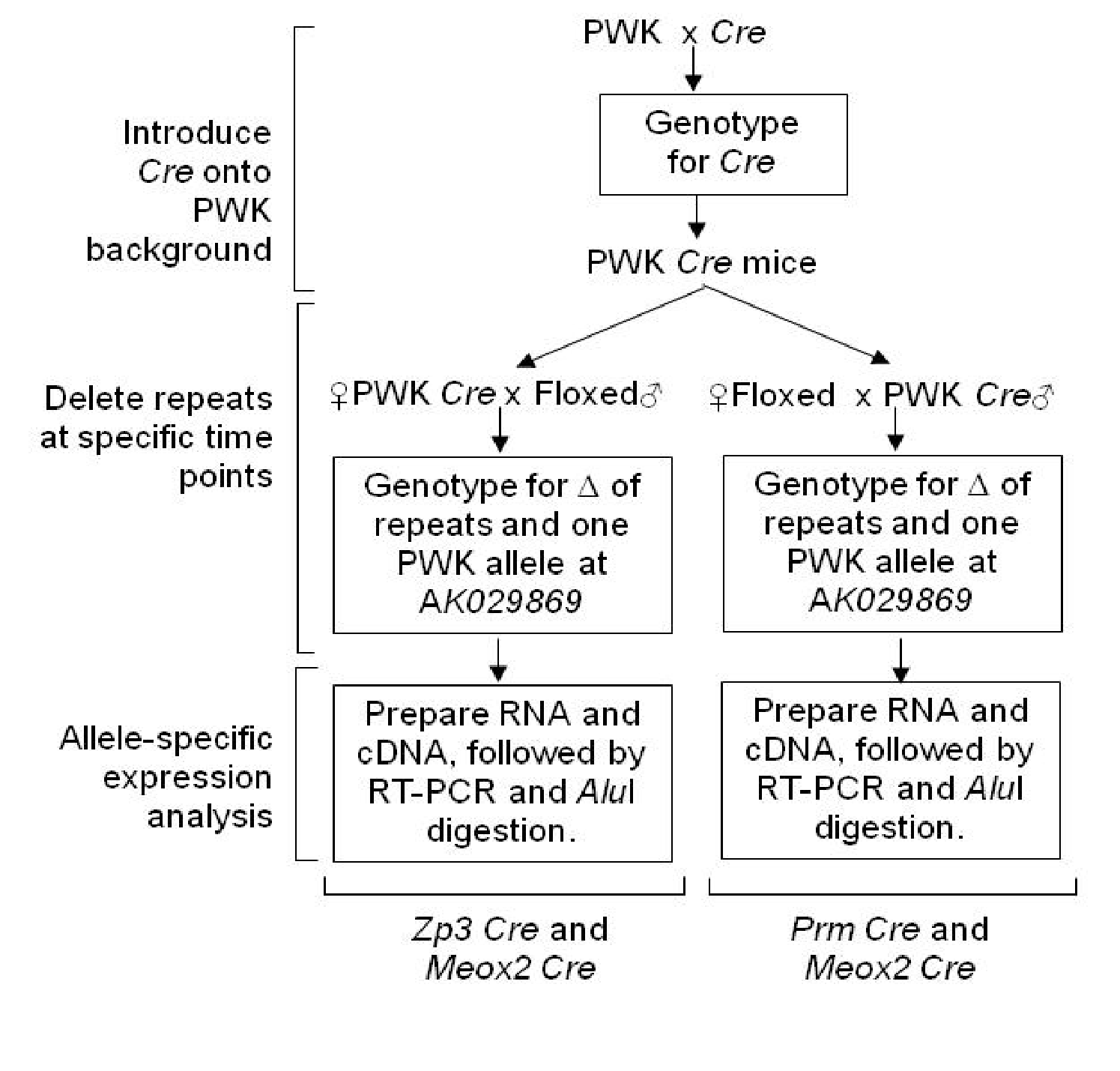

Supplement: Figure S1 — Crossing scheme for developmental time point specific repeat deletions. To delete the DNA repeats as specific times after fertilization, we used an allele containing a loxP-flanked copy of the Rasgrf1 DNA repeats in combination with the Cre transgenic mice. To facilitate allele-specific expression analysis, we bred specific Cre alleles onto the PWK/PhJ mouse background. These mice (PWK Cre) were mated with mice homozygous for a loxP-flanked version of the Rasgrf1 DNA repeats (Floxed), which was created on the 129S4Jae strain background and backcrossed to C67BL/6. In the presence of Cre recombinase expression, the loxP-flanked repeats can be deleted at specific time points. Zp3 Cre is active at e0.0 and deletes the repeats at the one-cell stage, while Meox2 Cre is active at e5.5 and deletes the repeats in the embryonic ectoderm of the e5.5 epiblast around the time of implantation into the uterine wall. Depending on whether the loxP-flanked repeats were inherited maternally or paternally, we were able to delete the repeats at these time points on either the maternally or the paternally inherited allele. After genotyping to ensure that the animals were not mosaic for deletion of the DNA repeats, and to ensure that the animals carried one PWK and one 129S4Jae allele at the AK029869 locus, we carried out allele-specific expression analysis. (0.42 MB TIF) [file pone.0013784.s001.tif]

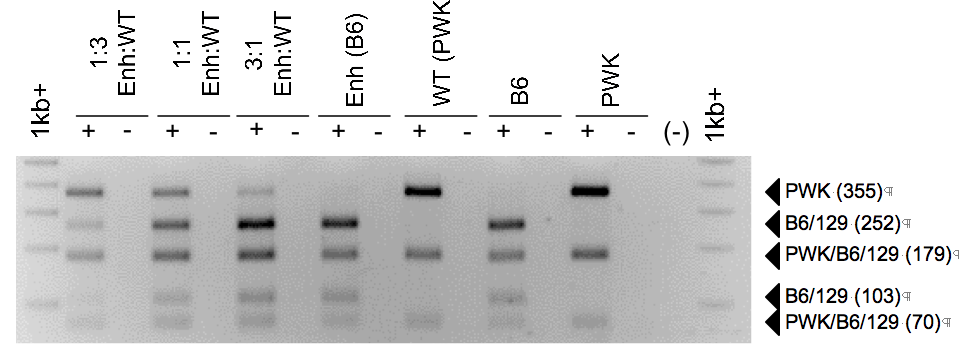

Supplement: Figure S2 — The presence of an extra enhancer allele does not mask detection of expression from wild-type alleles in trans. cDNAs from a wildtype and an extra enhancer allele were mixed in varying ratios. The mixed cDNAs were subjected to allele specific expression analysis of AK029869 as described in the main text. Strains and band sizes are shown to the right (see main text, Figure 2A). In each case, even when the extra enhancer allele (enh) was present in a 3∶1 ratio to the wild-type allele (WT), banding patterns from both alleles were present indicating that expression from the extra enhancer allele did not obscure expression from the wild-type allele during amplification. “ + ” = with reverse transcriptase, “ – ” = without reverse transcriptase, (-) = water, B6 = C57Bl6/J, PWK = PWK/PhJ. (0.17 MB TIF) [file pone.0013784.s002.tif]
